# Investigating Epigenetic and Neuroimaging Profiles in Bipolar Disorder and Behavioral Variant Frontotemporal Dementia: An integrated epigenetic-neuroimaging approach

**DOI:** 10.1192/j.eurpsy.2024.1296

**Published:** 2024-08-27

**Authors:** G. Delvecchio, M. Serpente, L. Di Consoli, E. Rotondo, V. Borraci, E. Scola, F. M. Triuzi, M. Castellani, A. Arighi, E. Scarpini, D. Galimberti, P. Brambilla

**Affiliations:** ^1^Department of Neurosciences and Mental Health; ^2^Department of Neuroradiology; ^3^Nuclear Medicine Unit, Fondazione IRCCS Ca’ Granda Ospedale Maggiore Policlinico; ^4^Department of Biomedical, Surgical and Dental Sciences; ^5^Department of Pathophysiology and Transplantation, University of Milan, Milan, Italy

## Abstract

**Introduction:**

Discriminating between bipolar disorder (BD) and behavioral variant Frontotemporal Dementia (bvFTD) is a clinical challenge as it is still based on clinical judgement, which often leads to misdiagnosis. This challenge is particularly pronounced in cases involving the *C9orf72* hexanucleotide expansion, a genetic factor responsible for a substantial portion of familial FTD cases, as in these patients the development of late psychoses is particularly frequent. Moreover, individuals with *C9orf72* bvFTD are also characterized by behavioral changes that resemble those seen in late-life BD, especially during the early stages of the disease. This raises questions about whether the clinical similarities between BD and bvFTD are rooted in specific alterations within the brain networks involved in cognitive processing or in selective genetic and epigenetic mutations. In light of this, our recently published neuroimaging study has shed light on the presence of distinctive structural and metabolic characteristics in elderly individuals with BD and bvFTD. These findings offer valuable neurobiological insights that may lead to differentiate between bvFTD and elderly BD patients.

**Objectives:**

Building on our previous research, this study further explores the existence of similar epigenetic expression patterns in plasma neural derived extra cellular vesicles (NDEs), such as miRNA and lncRNA, and seeks to correlate these epigenetic data with shared or distinct biological markers obtained through structural Magnetic Resonance Imaging and [18F]-fluorodeoxyglucose (FDG)-Positron Emission Tomography (PET).

**Methods:**

We will plan to conduct statistical analyses on epigenetic and neuroimaging data on *C9orf72* and sporadic bvFTD as well as on late-and early-onset BD patients and on healthy controls. Additionally, A PET study will be also performed on a subpopulation of these patients.

**Results:**

Our hypothesis posits that selective epigenetic modifications may impact the brain’s structure and function, in a way that can change the glutamatergic neurotransmission in prefrontal regions, with subsequent indirect effects on subcortical areas.

**Conclusions:**

Our findings will not only help identifying the specific biological signatures of BD and bvFTD, which might have important implications not only in prevention but also in differential diagnosis and treatment, but also offer insights into potential targets for slowing the onset and progression of the structural alterations characterizing these disorders.

**Disclosure of Interest:**

None Declared

